# Molecular Mechanisms Associated with Neurodegeneration of Neurotropic Viral Infection

**DOI:** 10.1007/s12035-023-03761-6

**Published:** 2023-11-09

**Authors:** Prapimpun Wongchitrat, Theerawut Chanmee, Piyarat Govitrapong

**Affiliations:** 1 Center for Research Innovation and Biomedical Informatics, Faculty of Medical Technology, Mahidol University, 999 Phutthamonthon 4 Road, Salaya, Phutthamonthon, Nakhon Pathom, 73170 Thailand; 2 Department of Clinical Chemistry, Faculty of Medical Technology, Mahidol University, Salaya, Nakhon Pathom, Thailand; 3 Chulabhorn Graduate Institute, Chulabhorn Royal Academy, Bangkok, Thailand

**Keywords:** Neurodegeneration, Viral infection, CNS infection, Neurotropic virus, Oxidative stress, Apoptosis, Autophagy, Mitophagy, Neuroinflammation, Neuronal metabolism, Neurotransmission system, Alzheimer’s disease

## Abstract

Viral infections of the central nervous system (CNS) cause variable outcomes from acute to severe neurological sequelae with increased morbidity and mortality. Viral neuroinvasion directly or indirectly induces encephalitis via dysregulation of the immune response and contributes to the alteration of neuronal function and the degeneration of neuronal cells. This review provides an overview of the cellular and molecular mechanisms of virus-induced neurodegeneration. Neurotropic viral infections influence many aspects of neuronal dysfunction, including promoting chronic inflammation, inducing cellular oxidative stress, impairing mitophagy, encountering mitochondrial dynamics, enhancing metabolic rewiring, altering neurotransmitter systems, and inducing misfolded and aggregated pathological proteins associated with neurodegenerative diseases. These pathogenetic mechanisms create a multidimensional injury of the brain that leads to specific neuronal and brain dysfunction. The understanding of the molecular mechanisms underlying the neurophathogenesis associated with neurodegeneration of viral infection may emphasize the strategies for prevention, protection, and treatment of virus infection of the CNS.

## Background

Neurodegeneration is the progressive atrophy and loss of function of neurons, glial cells, and the neural networks in the brain and spinal cord. This degeneration and death of neurons often leads to several neurodegenerative diseases that cause complex and serious medical conditions that worsen over time with motor, cognitive, and autonomic dysfunction, such as Alzheimer’s disease (AD), Parkinson’s disease (PD), Huntington’s disease (HD), amyotrophic lateral sclerosis (ALS), and prion diseases [1]. Different neuropathologic mechanistic processes cause neuronal death in neurodegenerative diseases including the oxidative stress due to the overproduction of free radicals with the decline of cellular antioxidant defense systems. Excessive free radical formation induces mitochondrial dysfunction, mitochondrial dynamic imbalance, and impaired bioenergetics which contribute to the redox imbalance that can cause malfunctioning of the endoplasmic reticulum (ER), resulting in the abnormal protein accumulation, calcium accumulation, and intrinsic cell death pathway activation [2]. Severe or prolonged stress activates cell death pathways, including autophagy and apoptosis [3]. Neuronal loss in neurodegenerative diseases is often associated with the deposition of extra- and intracellular misfolded proteins that are toxic to neurons, impair mitochondrial redox activity, and increases the generation of oxidative stress that activates apoptosis signaling to trigger cell death [4]. The activation of an immune response by microglia and astrocytes which occurs in response to the cytotoxic consequences of the aggregation of misfolded proteins induces the production of several inflammatory factors that promote chronic neuroinflammation associated with the progression of neuronal loss in the CNS [5]. These interrelated mechanisms are involved in the death and dysfunction of neurons in neurodegenerative diseases.

Neurotropic viruses are common causes of CNS infections, both acute and chronic viral neurologic syndromes, including meningitis, encephalitis, encephalomyelitis, and myelitis [6]. Viruses can enter the CNS via two major routes: blood circulation (viremia) and crossing the blood–brain barrier (BBB) or entering via peripheral nerve endings. Viral entry into the brain through the BBB can occur through three different mechanisms: the transcellular pathway (virus passage through infected endothelial cells), the “Trojan Horse” pathway (virus transport using infected immune cells), or the paracellular pathway (virus entry through disruption of junction proteins, the actin cytoskeleton, or the basal lamina) [7, 8]. The invasion of neurotropic viruses into the CNS is associated with neurodegeneration through a variety of mechanisms. Viruses primarily infect neurons through interaction with host attachment factors and receptors, followed by either direct fusion to the plasma membrane (enveloped viruses) or endocytosis (non-enveloped viruses) and subsequent delivery of the viral genome (DNA or RNA) to the cytosol or the nucleus of the infected cell [9]. Upon entry, the virus replicates within neuronal cells, and the accumulation of viral antigens induces an increase in oxidative stress which leads to the activation of both innate and adaptive immune responses. Viral components are recognized by host pattern recognition receptors (PRRs) such as Toll-like receptors (TLRs), C-type lectin receptors (CLRs), NOD-like receptors (NLRs), Retinoic acid-inducible gene-I (RIG-I)-like receptors (RLRs), and cytosolic DNA sensors (CDS) which are widely expressed in the CNS cells including microglia, neurons, astrocytes, oligodendrocytes, epithelial cells, and innate immune cells [7]. Activation of PRR signaling triggers the production of inflammatory mediators in order to clear viral invasion. However, chronic activation of these receptors can cause inflammatory damage [10]. Neurotropic virus infection induces neuronal damage through direct killing, cell lysis, increased free radical release, perturbation of the cellular stress response, cellular activation of neuroinflammation, and induction of apoptotic signaling leading to neuronal cell death [11, 12]. In addition, viruses can infect neuroglial cells, which results in the activation of astrocytes and microglia, leading to the production of numerous proinflammatory cytokines. The overproduction of proinflammatory cytokines induces an increase in the permeability of the BBB, which allows the virus to easily enter the CNS. Moreover, the increase in proinflammatory cytokines induces neuroinflammation, which leads to pathological changes such as cellular infiltration, perivascular cuffing meningeal disruption, neuronal shrinkage, and plaque formation in brain tissues. Viral infections disrupt the cellular function of neurons, such as metabolic pathways and neurotransmitter synthesis, which result in neuronal and brain dysfunctions. Viral infections implicate pathogenic etiology in neurodegenerative diseases. In this review, we provide and summarize the molecular mechanisms of neuropathogenesis associated with neurotropic virus-induced neurodegeneration.

## Viral Infection Induces Oxidative Stress

**Fig. 1:**
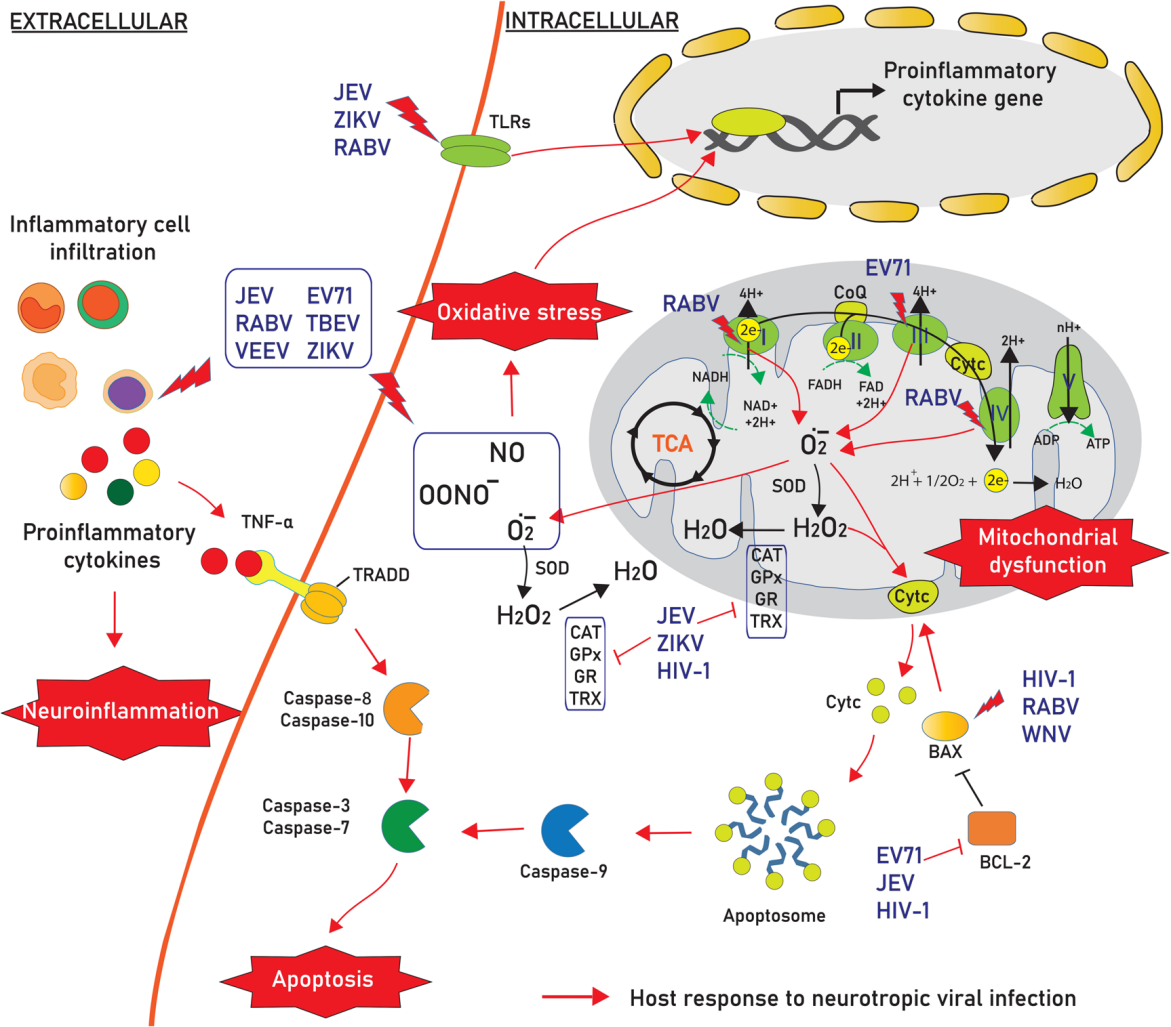
Neurotropic viral infection induces neurodegeneration through a variety of cellular mechanisms. Viral infection affects several host cell response mechanisms to attenuate neuronal functions, including (i) increasing reactive oxygen species (ROS) by interfering with the electron transport system and disrupting the production of antioxidants, (ii) promoting neuroinflammation by increasing proinflammatory cytokine secretion from infiltrated inflammatory cells and infected neuronal cells such as microglia and astrocytes, which then (iii) activates the apoptosis signaling pathway by inducing both intrinsic and extrinsic signaling pathways leading to neuronal death in the CNS. Abbreviation: Cyt c, cytochrome c; CoQ, coenzyme Q; BCL-2, B-cell lymphoma 2; BAX, BCL-2 associated X; FAD, flavin adenine dinucleotide; FADH, flavin adenine dinucleotide hydride; NADH, reduced nicotinamide adenine dinucleotide; NAD+, nicotinamide adenine dinucleotide; TNF-α, tumor necrosis factor alpha; TRADD, TNF receptor-associated death domain; CAT, catalase; SOD, superoxide dismutase; GPx, glutathione peroxidase; GR, glutathione reductase; TRX, thioredoxin; TLRs, toll-like receptors; TCA, tricarboxylic acid

Infection with neurotropic viruses promotes oxidative stress, which is associated with excessive production of reactive oxygen species (ROS) and insufficient cellular antioxidant defenses. The disturbance in the oxidant–antioxidant balance leads to potential cellular damage in the host cell. ROS can damage cell components such as nucleic acids, lipids, and proteins and subsequently disturb their functions, contributing to neurodegeneration conditions in the CNS. Mitochondria are the major source of intracellular ROS and antioxidant enzymes, which maintain redox balance. Neurotropic viruses causing both acute and slow infection can induce the generation of ROS, which can initiate the lipid peroxidation process and play a critical role in cellular death (Fig. 1).

Japanese encephalitis virus (JEV; family *Flaviviridae*) infection can cause Japanese encephalitis in humans with a high fatality rate in severe cases, and ~30–50% of survivors experience serious permanent neurologic sequelae or psychiatric sequelae [13, 14]. JEV infection increases the level of ROS production, which increases neuronal cell death triggered by both mature viruses and replication-incompetent virions [15]. ROS overproduction together with a decrease in membrane fluidity in JEV-infected neuronal cells causes severe cytopathic effects and subsequently contributes to neuronal cell death [16]. JEV infection increases the levels of superoxide anions (O2.-), nitric oxide (NO), and peroxynitrite (OONO-) in neurons and glial cells [17–19]. Excessive O2.- production during viral infection was also observed in neuronal cells infected with other members of *Flaviviridae* family, including West Nile virus (WNV) [20] and dengue virus type 2 (DENV-2) [21], leading to host cell apoptosis.

Venezuelan equine encephalitis virus (VEEV; family *Togaviridae)* causes severe zoonotic disease in humans, and approximately 4–14% of cases develop serious neurological complications and 1% develop lethal encephalitis [22–24]. VEEV infection significantly increases in ROS levels in astocytoma U87MG cells [25]. An increase in NO formation together with the activation of inducible nitric oxide synthase (iNOS) was observed in the brains of mice infected with VEEV [26]. The overproduction and activation of malondialdehyde (MDA), a lipid peroxidation marker, was detected in the brains of JEV-infected animals [27]. Lipid peroxidation induced by VEEV has been shown to increase the concentration of thiobarbituric acid reactive substances (TBARS), a byproduct of lipid peroxidation, in the mouse brain [26].

Rabies virus (RABV; family *Rhabdoviridae*) is a deadly virus that infects the CNS and causes encephalitis, ultimately resulting in death in mammals [28]. RABV infection is shown to increase the ROS production in mouse neuroblastoma cells [29]. The viral component of RABV plays a critical role in the induction of oxidative stress [30]. Immunostaining for 4-hydroxy-2-nonenal (4-HNE) indicated evidence of lipid peroxidation associated with oxidative stress that causes axonal injury, as shown by axonal swelling and reduced axonal growth in the dorsal root ganglion of RABV-infected mice [31]. Another member of the *Rhabdoviridae* family, Chandipura virus (CHPV), an enveloped RNA virus that causes acute encephalitis mainly in children, has been reported to induce neuronal apoptosis by stimulating oxidative stress. CHPV infection increases the intracellular Ca^2+^ secretion, which further increases ROS, superoxide production levels, and mitochondrial dysfunction within CHPV-infected cells and causes neuronal death *in vitro* and *in vivo* [32–34].

Enterovirus 71 (EV71; family *Picornaviridae*) is a major causative agent of hand, foot, and mouth disease (HFMD) with fatal neurological complications in young children. EV71 infection can lead to increased ROS generation and activation of nicotinamide adenine dinucleotide phosphate (NADPH) oxidase, which in turn enhances EV71 infection in neural cells [35–37].

Oxidative stress is also seen in viruses causing latent or slow infections, such as herpes simplex virus (HSV; family *Herpesviridae*) and human immunodeficiency virus (HIV; family *Retroviridae*). Herpes simplex encephalitis is caused by herpes simplex virus type 1 (HSV-1) and is a common cause of sporadic focal encephalitis worldwide [38]. Intracellular ROS generation in response to HSV-1 infection was observed in microglia [39] and neural cells [40]. HSV-1 infection causes oxidative stress and induces the release of bioactive lipid peroxidation byproducts, MDA/hydroxyalkenals (HAEs), in cultured mouse neural cells, which is necessary for virus replication [40]. HIV causes immunodeficiency, which leads to acquired immunodeficiency syndrome (AIDS). HIV infects the CNS and enhances neurotoxicity, which directly harms the brain and manifests as HIV-associated neurocognitive disorders (HANDs) [41–43]. Several component proteins of human immunodeficiency virus type-1 (HIV-1) enhance ROS production in neuronal cells, including neurons, microglial cells, and astrocytes, by different mechanisms [44–46]. The HIV-1 transactivator of transcription (Tat) protein induces the production of ROS and significantly induces DNA breakage [47]. Increased levels of nitroxidative stress marker proteins such as NADPH oxidase, cytochrome P450-2E1 (CYP2E1), and iNOS are observed in HIV-1 transgenic rat brains [48].

ZIKV targeting of neuronal cells causes neurological complications such as congenital microcephaly, Guillain–Barré syndrome, transverse myelitis, and meningoencephalitis [49, 50]. ZIKV impairs mitochondrial structure and function, as shown by the decrease in oxygen flux coupled with adenosine triphosphate synthesis [51]. ZIKV infection leads to the increased production of ROS, which is associated with DNA breakage in neural cells *in vivo* and *in vitro* [51, 52]. ZIKV infection resulted in a significant increase in lipid peroxidation, as observed by the high levels of MDA and carbonyl protein in human glioblastoma-infected cells [52].

The increase in free radicals and lipid peroxidation induced by neurotrophic viruses demonstrates that oxidative stress contributes as a key factor in the pathogenesis of neurodegeneration in viral infection of the CNS.

## Virus Infection Disturbs the Production of Antioxidants

The elevation of cellular oxidative stress due to increases in free radicals and lipid peroxidation was associated with an opposite decrease in antioxidant activities. Viral infections stimulate ROS production and inhibit antioxidant enzyme levels [53, 54] (Fig. 1). The antioxidant defense mechanism is impaired by a decline in intracellular antioxidant levels, such as superoxide dismutase 1 (SOD-1), thioredoxin 1 (TRX-1), and glutathione (GSH), and a reduction in catalase (CAT) and glutathione peroxidase (GPx) activities in neuronal cells and several brain regions during JEV infection [16, 27]. EV71 diminished the ratio of GSH to its disulfide form, glutathione disulfide (GSSG), an indicator of oxidative stress [55]. ZIKV-induced oxidative stress is correlated with decreased levels of SOD and CAT activities in U87-MG cells and the brains of C57BL/6 mice [52]. Infection with HSV-1 has been reported to induce the depletion of GSH [56]. Exposure of rat brain endothelial cells to HIV-1 envelope glycoprotein GP120 (gp120) and Tat significantly decreases the levels of intracellular GSH, GPx, and glutathione reductase (GR) and the ratio of GSH/GSSG [57]. The decrease in antioxidant enzyme activities after ZIKV infection was found to be associated with the negative regulation of nuclear factor erythroid 2-related factor 2 (NRF2)/antioxidant response element (ARE) signaling [52]. This evidence suggests that depletion of the antioxidant enzymatic system occurs during viral infection.

## Virus Infection Induces Mitochondrial and Endoplasmic Reticulum Stresses

Mitochondrial oxidative stress is involved in pathological statuses and is the major source of excessive amounts of ROS in infected cells. ROS accumulation in cells is a direct reflection of disruption in mitochondrial electron transport chain (ETC) function and redox imbalance in the ER lumen, leading to the accumulation of unfolded proteins and in turn increasing oxidative stress, which results in increased cellular damage and apoptosis. An increase in ETC-related protein activity was observed after viral infection (Fig. 1). RABV infection significantly alters a variety of mitochondrial parameters, such as increases in maximal uncoupled respiration and complex IV respiration and mitochondrial complex I and complex IV activities in neurons [58]. The activity of mitochondrial complex I, a site of ROS production, was correlated with the high level of ROS in mouse neuroblastoma cells [29]. The increase in the generation of ROS and oxidative stress caused by the specific 139–172 region of the RABV phosphoprotein that interacts with complex I in mitochondria causes mitochondrial dysfunction [29]. RABV infection induces mitochondrial ETC dysfunction, resulting in oxidative stress and degenerative changes in neuronal processes (involving both dendrites and axons) in infected mice [31, 59, 60]. Experiments using inhibitors of several mitochondrial proteins showed that EV71 mainly induces ROS generation at a site of the mitochondrial ETC distal to mitochondrial complex III [35]. Increased mitochondrial oxidative stress by EV71 infection can cause mitochondrial morphological changes and exhibit functional anomalies, such as a decrease in mitochondrial electrochemical potential and a lower respiratory control ratio of mitochondria in glioblastoma cells [35].

Together with mitochondrial oxidative stress, ER stress usually occurs simultaneously to generate cellular stress in virus-infected cells. The ER is responsible for the correct three-dimensional conformation of protein folding and maturation. The formation of disulfide bonds produces large amounts of ROS and depletes GSH, contributing to the redox imbalance that can cause malfunctioning of the ER, including massive protein production, loss of Ca^2+^ homeostasis, inhibition of N-linked glycosylation, and accumulation of mutant proteins [61, 62]. Viral replication can cause an increase in viral protein synthesis demand to overcome the ER folding capacity, leading to the massive production of misfolded viral proteins that accumulate in the ER lumen and trigger ER stress [63]. JEV infection induces ER stress as evidence of the detection of the excessive proliferation of ER membranes together with the amount of viral proteins and the induction of unfolded protein response (UPR) signaling in neuronal N18 and NT-2 cells [16]. In human neural stem cells (hNS1 cells), JEV infection promoted the expression of ER stress-related proteins such as glucose-regulated protein 78 kDa (GRP78), heat shock protein (HSP) 60, HSP70, and HSP90 [64]. JEV infection activates several ER stress sensors, including protein kinase RNA-like ER kinase (PERK), activating transcription factor 4 (ATF4), and C/EBP homologous protein (CHOP) under acute or prolonged ER stress. The JEV-induced UPR provokes CHOP/growth arrest and DNA damage-inducible protein (GADD153) and triggers the activation of p38 mitogen-activated protein kinase (MAPK), which enhances JEV-induced apoptosis via the activation of the caspase cascade [16]. JEV nonstructural protein 4B (NS4B) activates the PERK/ATF4/CHOP neuronal apoptosis pathway both *in vitro* and *in vivo* [65].

WNV infection activates multiple branches of ER stress-mediated UPR pathways, leading to transcriptional and translational induction of UPR target genes [63]. Activation of ATF6 and PERK pathways was induced during WNV infection in SK-N-MC neuroblastoma cells, resulting in CHOP activation and downstream apoptosis [66]. The transcriptomic analysis shows that the components of the UPR-related genes such as *PERK*, *ATF4*, and *DDIT3* (encoded CHOP) and early growth response 1 (*EGR1*) are activated in VEEV-infected human astrocytoma cells [67]. The activation of EGR1 is regulated by extracellular-signal-regulated kinase (ERK) and PERK pathways, which are important in contributing to neural cell death in VEEV infection [68].

ZIKV infection activates ER stress by significantly increasing the expression of ER stress markers in neural cells [69]. ZIKV upregulates UPR-related genes in the cerebral cortex of infected postmortem human fetuses as well as in cultured human neural stem cells and in the mouse embryonic brain [70]. Transfection with EV71 viral envelope protein 1 (VP1) increased translation initiation factor 2α (eIF2α) kinase phosphorylates Serine51 (Ser51) phosphorylation, which represented to ER stress activation in mouse brainstem neurons [71]. In mouse Schwann cells, the overexpression of VP1 also induced ER stress, leading to the upregulation of peripheral myelin protein 22 (PMP22) [72]. These observations indicate the role of ER stress in the pathogenesis induced by viral infection.

## Virus Infection Induces Alteration of Autophagy

The cellular stress response induced by virus infection is also related to the induction of autophagy in infected cells [73]. CHPV infection shows an increase in microtubule-associated protein 1 light chain 3 beta (LC3B), an autophagic marker, associated with overproduction of ROS and mitochondria dysfunction in HT-22 mouse hippocampal neuronal cell [32]. Infection with EV71 shows an increase in LC3-II protein as well as the formation of LC3 aggregates, autophagosomes, and amphisomes in several infected brain tissues of mice [74, 75]. Immunofluorescence staining indicated the colocalization of EV71 proteins with LC3 and mannose-6-phosphate receptor (MPR, endosome marker) proteins, which indicates amphisome formation accompanied by autophagic flux in EV71-infected SK-N-SH cells [74]. The EV71 VP1 protein is an important neurovirulence protein that induces autophagy by regulating the mammalian target of rapamycin (mTOR) signaling pathway to promote viral replication [75–77]. Phosphorylated mTOR, phosphorylated 70-kDa S6 kinase (p70S6K), and peroxisomal acyl-coenzyme A oxidase 1 (ACOX1) are signaling pathways involved in EV71-induced autophagy and neural cell apoptosis when downregulated [36, 75, 76]. In HIV-1 associated encephalitis, increases in several autophagic markers, such as Beclin-1, autophagy-related gene (Atg)-5, Atg-7, and LC3-II, have been observed in frontal cortex postmortem brains [78]. The SK-N-SH cells treated with gp120 from C-X-C motif chemokine receptor 4 (CXCR4) and C-C motif chemokine receptor 5 (CCR5)-tropic HIV-1 virus exhibit the accumulation of autophagic proteins and autophagosomes [78]. Autophagy in HAND has also been evaluated. HIV-1 proteins such as Nef and Tat alter neural autophagy in different manners related to the autophagosome formation and autophagic flux, which may contribute to HAND [79–81]. The JEV-activated autophagy has been explored in both N2a neuroblastoma cells and mouse brains, and it demonstrated an increase in LC3-II protein accumulation and induction of autophagosome formation [82].

## Virus Infection Induces Impairment of Mitophagy and Mitochondrial Dynamics

In addition, viruses induced impairment of mitochondrial dynamics and triggered mitophagy, a specific autophagy form for the removal of damaged mitochondria, directly and indirectly, and controlled the mitophagic process via different strategies [83]. The accumulation of ROS by VEEV TC-83 infection alters mitochondrial dynamics by affecting the expression of dynamin-related protein 1 (DRP1), a protein that plays a role in mitochondrial fission, resulting in an increase in mitochondrial fractions in infected astrocytes [25]. PTEN-induced kinase 1 (PINK1) and Parkin, proteins associated with mitophagy, appear to be enriched in mitochondrial fractions, indicating that mitochondrial damage contributes to the apoptosis of infected cells [25]. As shown in human primary neurons, HIV-1 gp120 and Tat proteins induce the mitochondrial fission process via DRP1 as a result of neural mitochondrial fragmentation and then activate mitophagy markers such as LC3B and Beclin-1 and recruit PINK and Parkin sequestosome 1 (SQSTM1) to damaged mitochondria [84–87]. However, HIV-1 proteins are found to inhibit mitophagic flux in human primary neurons by impairing the delivery of mitochondria to the lysosomal compartment, leading to incomplete neuronal mitophagy, which causes neuronal damage [84]. Dysregulation of autophagy and mitophagy and alteration of mitochondrial dynamics may be important and contribute to the pathogenesis of neurotropic virus infection.

## Virus Infection Activates the Apoptosis Signaling Pathway

Oxidative stress elicits the loss of mitochondrial membrane potential and results in the induction of the intrinsic apoptosis pathway [16, 88, 89]. The expression of apoptotic protein markers has been observed in several neurotropic virus-infected cells (Fig. 1). Overexpression of caspase-3 is observed in the brains of pediatric patients with HIV-1 encephalitis and corresponds to increases in DNA fragmentation, a marker of apoptotic cells [90]. In the HIV-1 model, the levels of BCL2 associated X (BAX) and activated caspase-3 were significantly elevated in the hippocampus and were associated with neuronal cell death in HIV-1 transgenic rats [48]. HIV-1 gp120 protein induces apoptosis in neurons and microglial cells in association with the activation of MAPK pathways mediated by ERK and JNK and lowering the expression of B-cell lymphoma 2 (BCL-2) [42, 91, 92]. BCL-2 is a major target of HIV-induced changes that are modulated to different degrees during HIV infection, resulting in either a proapoptotic or an antiapoptotic phenotype [93]. VEEV infection causes neuronal injury ranging from nuclear chromatin condensations to nuclear and cellular fragmentation, indicating apoptotic cell death [94]. The positron emission tomography (PET) with a tracer targeting the caspase-3 substrate revealed an increase in apoptosis and a decrease in BBB integrity in VEEV-infected mouse brains [95]. EV71 markedly reduces BCL-2 expression but induces an increase in the mRNA expression of several apoptosis-promoting factors, such as *BAX*, *CASP7* (caspase-7), *CASP3* (caspase-3), and cleaved caspase-3 [96]. EV71 infection triggers the translocation of cytochrome c (Cyt c) from mitochondria to the cytosol, and caspase-9 is activated, leading to neural cell death and indicating that the mitochondria-mediated intrinsic apoptotic pathway is activated by EV71 [97, 98]. EV71 also induces cell cycle arrest of SH-SY5Y cells through stimulation of endogenous microRNA let-7b expression after EV71 infection [96]. The invasion of RABV into the CNS shows morphologic changes of apoptosis and marked increases in BAX in the hippocampus and cerebral cortex [99–103]. The marked apoptotic are present in neurons, glial cells, and perivascular mononuclear cells within the white matter of the cerebellum of dogs with the positive detection of BCL-2 and BAX protein [104]. The morphological changes and apoptosis of spinal neurons and dorsal root spinal ganglion cells are present in the late period of infection [105]. The replication of RABV in mouse neuroblastoma cells increases the level of BAX and caspase activation, which induces the degradation of poly ADP-ribose polymerase (PARP), leading to destruction of the DNA [106]. WNV proteins such as capsid and nonstructural protein 3 (NS3) induce apoptotic features in CNS cells via BAX-dependent apoptosis that triggers mitochondria-outer-membrane-permeabilization [107–111]. WNV replication decreased cell viability and induced upregulation of BAX expression and the release of Cyt c from the mitochondria and formation of apoptosomes, followed by the activation of the effector caspase-3, the initiator caspases-8 and -9 then cleavage of the PARP in which induces neuronal cell death [66, 108, 111–114]. JEV infection promoted increased the expression of BAX [89, 115], cleaved PARP levels [88], and the activation of caspase-3 activity [116], were observed in JEV-infected cells (Fig. 1). JEV infection also increased Cyt c release and caspase 3 activation in cultured neuronal cells and in infected mouse brain, inducing neuronal death, and increasing the mortality rate of mice [19, 115, 117]. A reduction in BCL-2 expression levels was found in JEV-infected neuronal cells and the mouse brain [115]. Several studies have also reported the overexpression of BCL-2 levels at the early stage of JEV infection, which helps to the induce host cell survival in order to facilitate viral persistence [117–121]. However, the studies showed that the increase in BCL-2 during JEV infection failed to inhibit the infected cells from undergoing apoptosis and failed to block viral replication [117, 119, 121, 122]. Moreover, our recent study with a high multiplicity of infection (MOI) of Beijing-1 infection remarkably induced neuronal cell death via inducing the proteolysis of endogenous p21 BAX to generate more apoptogenic molecules of p18 BAX during the late stage of JEV infection [117]. The accumulation of cleaved p18 BAX might be due to the activation of calpain protease, which increases the intrinsic cytotoxic properties of this proapoptotic molecule and effectively induces Cyt c release from mitochondria, leading to the activation of the caspase cascade, which results in the induction of intrinsic apoptosis [123]. Thus, the intrinsic apoptosis pathway plays an important role in the pathogenesis of neurotropic virus infection.

## Virus Infection Induces Neuroinflammation

Neuroinflammation is an important feature of neurodegenerative disorders. Normally, neuroinflammatory processes play a protective role in response to CNS injury by several factors, including viral infections. Neurotropic viral infection of the CNS can cause the activation of microglia and astrocytes, inducing the production of several neuroinflammatory factors that promote chronic inflammation and increasing the progression of CNS diseases by the virus [124].

Inflammation is a hallmark of virus encephalitis. During JEV infection, various peripheral immune cell types infiltrate the CNS and affect the integrity of the BBB, which is a major regulator of neuroinflammation and viral propagation into the brain [125, 126]. In addition, peripheral immune cells infiltrating brain-resident cells interact with JEV upon infection of the brain. Microglial cells are the resident macrophages of the CNS, can be productively infected by JEV, and might serve as a reservoir for the virus [127]. The activation of microglia has been proposed to play a major role in neuronal cell death through the release of proinflammatory mediators [125, 128, 129]. The upregulation of TLR3, TLR7, and RIG-I signaling, including ERK, p38MAPK, activator protein 1 (AP-1), and nuclear factor kappa B (NF-κB), are triggered by JEV infection and induces the activation of microglia [116, 129] (Fig. 1). JEV induces encephalopathy by activating microglial cells and astrocytes that can increase the levels of proinflammatory cytokines such as tumor necrosis factor alpha (TNF-α), interleukins (IL-6, IL-8 and IL-10), and RANTES (regulated on activation, normal T-cell expressed and secreted) [130, 131].

Overproduction of chemokines and proinflammatory cytokines are key factors that play an important role in the pathogenesis of flavivirus infections. In the brain, the release of chemokines regulates the migration of leukocytes from the peripheral into the site of infection. Both clinical and experimental models suggest the involvement of neuroinflammation in dengue virus disease [132]. Intracranial injection of DENV is associated with the induction of interferon-beta (IFN-β), interferon-gamma (IFN-γ), TNF-α, chemokine (C-C motif) ligand 2 (CCL-2), CCL-5, C-X-C motif chemokine (CXCL)1, CXCL2, and IFN-stimulated gene (ISG) expression including viperin, Ifi27l2a, IRF7, and CXCL10 [133, 134]. DENV-2 and DENV-3 infections enhance of the infiltration of CD8+ and CD4+ T cells and neutrophils in the brain [132–134]. High levels of chemokines and proinflammatory cytokines such as IL-6, IL-1β, IFNγ, and TNF-α were associated with the severity of neuronal damage and the high mortality rate of mice during JEV infection [19, 131, 135–137]. TNF-α has a major effect on the induction of neuronal cell death in JEV infection by inducing the extrinsic apoptosis pathway via TNF receptor-associated death domain (TRADD) [89] and triggering inflammatory cells to release other cytokines, resulting in neuronal cell death [19, 131]. Moreover, IL-6, CXCL10, CCL-2, and CCL-5 downregulated the expression of BBB tight junction proteins, causing an increase in BBB permeability [136]. In addition, IL-1β activates the expression of adhesion molecules on epithelial cells, leading to inflammatory cell infiltration and the subsequent generation of numerous proinflammatory mediators, which ultimately cause neuronal cell death and irreversible brain damage [131, 136].

In another encephalitis virus, VEEV, PET tracer was used to target 18-kDa translocator protein (TSPO) to study the accumulation of macrophages and microglia/astrocyte activation; the results demonstrated an increase in neuroinflammation in the cortex, thalamus, striatum, hypothalamus, hippocampus, olfactory bulb, brain stem, and cerebellum of VEEV, and a decrease in BBB integrity was observed in VEEV-infected mouse brains [95]. VEEV-infected microglia produce several proinflammatory cytokines as a result of direct infection, including IFNγ, IL-1α, IL-1β, IL-6, and IL-12 [138].

The secretion of the proinflammatory cytokines, IFNγ, IL-6, and IL-1β in the cerebrospinal fluid (CSF) was increased in the acute stage of CNS involvement from EV71 infection [139, 140]. The histological studies of EV71-infected autopsy tissues showed obvious inflammation in the spinal cord gray matter, brainstem, hypothalamus, and subthalamic and dentate nuclei with perivascular cuffs, variable edema, neuronophagia, and microglial infiltration [141, 142]. EV71 infection increases TNF-α, IL-1β and RANTES production, which triggers bystander damage to neurons involving the tyrosine kinase/MAPKs/NF-κB signaling cascade during EV71 infection [143].

Tick-borne encephalitis virus (TBEV; family *Flaviviridae*) is an important tick-transmitted virus that causes tick-borne encephalitis (TBE) in Eurasia [144]. Increased levels several cytokines and chemokines have been detected in CSF or serum samples from TBE patients with higher ratios of IL-12:IL-4 and IL-12:IL-10, reflecting the global pro-inflammatory cytokine balance [145–148]. TBEV disrupts the BBB and infects neurons, astrocytes, and oligodendrocytes, inducing neuroinflammation followed by neuronal death [149–151]. TBEV infection upregulates the expression of several pathogen recognition receptors, pro-inflammatory cytokines, and interferon-stimulated genes in neuronal/glial cells [152, 153]. TBEV-infected mice exhibited time-dependent increases in serum and brain tissue concentrations of multiple cytokines/chemokines such as CXCL10/IP-10, CXCL1, G-CSF IL-6, and RANTES [153, 154]. Thus, neurons and astrocytes are potential sources of pro-inflammatory cytokines in TBEV infection (Fig. 1).

The CNS is the major target of ZIKV infection. In addition to the induction of massive neuronal cell damage, ZIKV-mediated neuroinflammation is the key to causing neurological pathology via an excessive inflammatory response, particularly in neonates. The relationship between ZIKV-induced neuroinflammation and postnatal microcephaly has been revealed in animal models and fatal cases of ZIKV-associated microcephaly [155, 156]. Brain histology demonstrated a pattern of inflammation, such as microglial activation, astrogliosis, vascular edema, lymphocytic infiltration, neuronal necrosis, neuronophagy, calcifications and apoptosis, which disrupted neural progenitor cells (NPCs) and neurovascular developments [155–157]. During ZIKV infection, activated microglia and astrocytes are mainly responsible for the production of several proinflammatory mediators, which are correlated with high expression of apoptosis markers in the brain and leakage of the BBB [155, 156, 158]. The molecular mechanism of ZIKV-mediated inflammation has been explored *in vitro* and *in vivo* [158–160]. TLR3 signaling was induced by ZIKV infection in both microglia and astrocytes, leading to increased NF-κB and PERK phosphorylation, which triggered the high production of several proinflammatory cytokines involved in the inflammatory process, such as IL-6, IL-1α, IL-4, IL-10, IL-8, monocyte chemoattractant protein-1 (MCP-1), RANTES, IFN-β, and transforming growth factor beta (TGF-β) [158] (Fig. 1). In addition, overproduction of IL-6, macrophage inflammatory protein (MIP)-1α, MIP-1β, MCP-1, TNF-α, IL-1Rα, IL-1α, IL-1β, IL-8, and IL-12p70 was observed in ZIKV-infected primary human fetal brain cells [160]. ZIKV infection induced the activation of NLR family pyrin domain containing 3 (NLRP3) inflammasome in glioblastoma cells [159]. Therefore, the brain damage by uncontrolled inflammation is an important pathogenesis of ZIKV infection.

In addition, in the RABV-infected brain, microglial cells and astrocytes were significantly increased in the areas of the nerve cells that showed apoptosis [100, 103, 161, 162]. The neuronal damage from RABV infection is caused by the high production of inflammatory cytokines in primary astrocytes and microglia [163]. The induction of inflammation in the mouse brain by RABV is observed by the overexpression of TLR3, TLR4, MIP-1α, RANTES, IP-10, MCP-1, TNF-α, and IL-6, which increases RABV pathogenicity [162, 164]. These observations demonstrate that the persistent and high-level expression of chemokines, excessive infiltration, and accumulation of inflammatory cells in the CNS, and severe enhancement of BBB permeability are the major features associated with the neuropathology by neurotropic infection.

## Viral Infection Alters Brain Metabolism

Over the past decade, it has become clear that viruses rely on host cell machinery to facilitate their replication. One of these mechanisms is based on the alteration of host cell metabolism, including glycolysis, the pentose phosphate pathway (PPP), the tricarboxylic acid (TCA) cycle, and oxidative phosphorylation, to facilitate optimal viral propagation. In the brain, it is probably more important to acknowledge that virus infection affects neuron and astrocyte metabolism, which disrupts brain function.

### Virus Infection Disturbs Neuron Metabolism

Brain development requires a controlled cellular metabolism for neural stem cell proliferation, differentiation, and maturation. Neural stem cell proliferation appears to have a specialized metabolism and is more dependent on glycolysis than oxidative phosphorylation for energy production. In contrast, neuronal differentiation is strongly manipulated by metabolic rewiring from glycolysis to oxidative phosphorylation [165]. Increasing evidence indicates that ZIKV infection suppresses neuron stem cell proliferation and induces premature differentiation, causing microcephaly of the newborn during pregnancy [166–168].

**Fig. 2:**
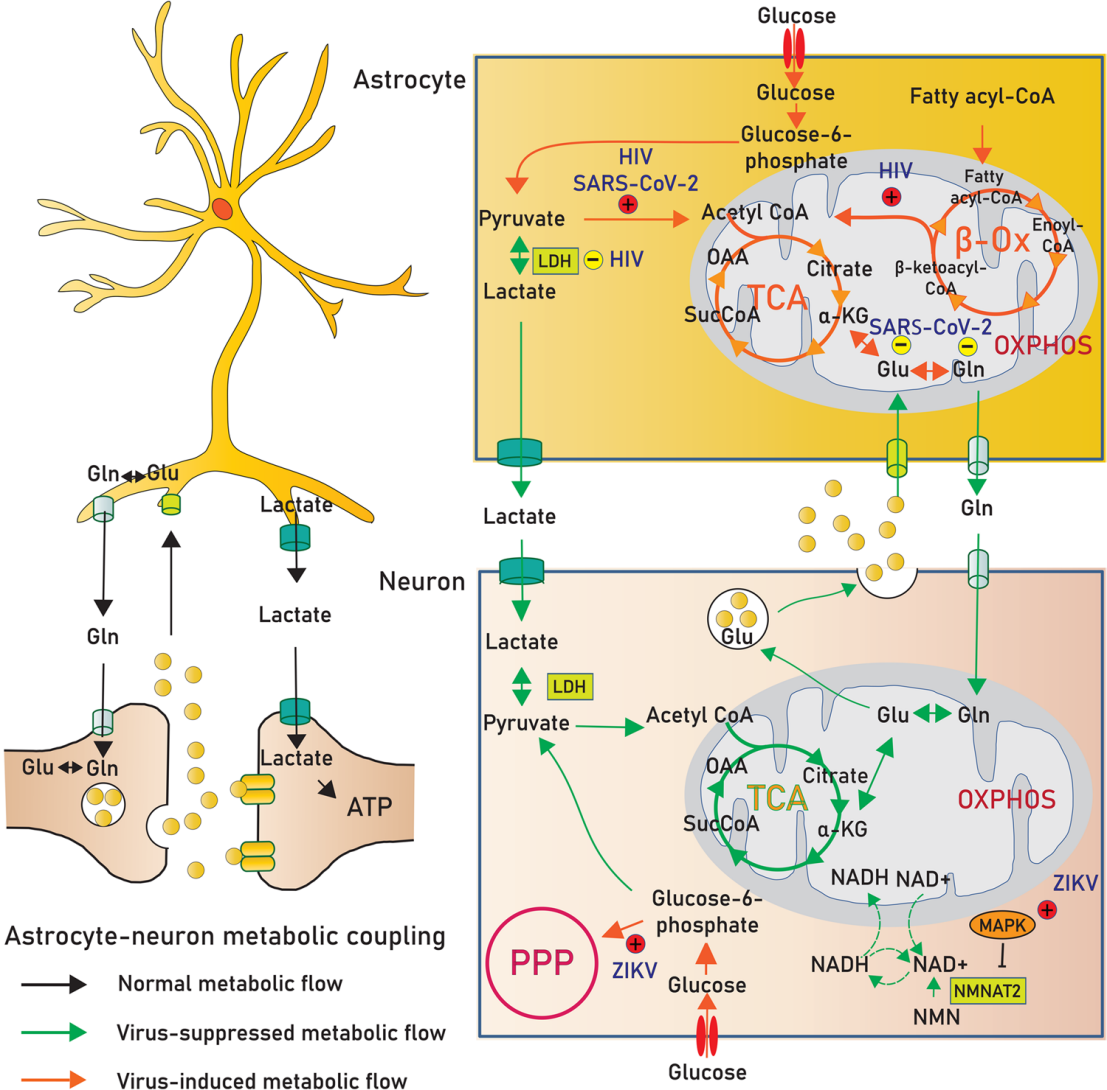
Cellular metabolism in the brain is altered by neurotrophic virus infection. Upon infection, there is a systemic metabolic alteration in neuron and astrocyte. The astrocyte to neuron lactate shuttle is inhibited and subsequently enhanced TCA cycle and oxidative phosphorylation in astrocytes. To fuel active TCA cycle, virus may activate fatty acid β-oxidation and glutamine metabolism. Within the neuron, the metabolic flow of glycolysis, TCA cycle, and oxidative phosphorylation is disrupted and selectively increased pentose phosphate pathway to sustain viral replication. Reduction of lactate production from astrocyte and dysregulation of neuron metabolism cooperatively induce neuron dysfunction. Abbreviation: α-KG, alpha ketoglutarate; β-Ox, beta oxidation; Gln, glutamine; Glu, glutamate; LDH, lactate dehydrogenase; NMN, nicotinamide mononucleotide; PPP, pentose phosphate pathway; OAA, oxaloacetate; OXPHOS, oxidative phosphorylation; SucCoA, succinyl-CoA; TCA, tricarboxylic acid

In recent publications, a multiomics study comprising the combination of metabolomic data with the information from transcriptomics and proteomics has been used to investigate and model the role of ZIKV-induced microcephaly. It demonstrated the metabolic alterations of nicotinamide adenine dinucleotide (NAD+)-related pathways including the TCA cycle, amino acid metabolism, and mitochondrial oxidative phosphorylation [169]. The mechanisms governing ZIKV disturbance of host cell metabolism are not yet fully understood. An activation of MAPK and cyclic guanidine monophosphate (GMP)-protein kinase G signaling upon ZIKV infection likely has a critical impact on the pathogenesis of ZIKV-induced microcephaly [169]. Initial studies demonstrated that NAD+ metabolism may be highly dependent on MAPK-mediated nicotinamide mononucleotide adenylyltransferase 2 (NMNAT2) degradation, thereby promoting axon degeneration [170, 171]; this suggests that ZIKV infection alters cellular metabolism via the MAPK-NMNAT2-NAD+ axis (Fig. 2). These signatures of metabolic reprogramming are consistent with the findings that ZIKV in both wild-type and mutant strains disrupts glycolytic flow into the TCA cycle, leading to mitochondrial dysfunction, which triggers inflammation and neuronal cell death [172]. In addition, due to the reduction in glycolysis and the TCA cycle seen in ZIKV-infected host cells, there is a dependence on the PPP (Fig. 2). For example, there is actually an increase in metabolic intermediates, such as guanidine diphosphate and GMP, in the PPP of ZIKV-infected cells [172]. Trends toward a decreased glucose contribution to the TCA cycle and an increased contribution to PPP cells to sustain viral propagation were also observed in infected C6/36 mosquitoes [173], suggesting that ZIKV infection in neurons may divert the carbon substrate into the PPP to promote viral replication.

### Virus Infection Disrupts Astrocyte-Neuron Metabolic Cooperation

The metabolism of neurons is functionally linked with astrocytes that provide energetic support to fuel the active neuron. While neuronal cells have robust aerobic glycolysis by converting glucose into acetyl-CoA for the production of substrates for the TCA cycle and oxidative phosphorylation, astrocytes, despite the presence of oxygen, favor the production of lactate. Astrocytes utilize glucose as their main energy source, and approximately 60% of glucose is converted to lactate [174], which is constitutively released to the extracellular milieu and taken up by neuronal cells to supply their high metabolic demand. This cooperative function of astrocytes and neurons is known as the astrocyte-to-neuron lactate shuttle (ANLS) [175]. Astrocyte-neuron metabolic cooperation is tightly regulated, but disrupting this ANLS by viral infection may cause abnormalities in brain function.

Neurological complications in HIV are strongly associated with cognitive impairment. Several studies have shown that induction of viral protein accelerates cellular damage in the CNS. HIV Tat release from infected cells can activate astrocytes and damage surrounding neurons [176, 177]. In fact, a number of studies have provided details about the potential mechanism underlying the role of HIV Tat in astrocyte metabolic shift and neurotoxicity (Fig. 2). First, the decrease in lactate dehydrogenase activity in Tat-activated astrocytes led to a reduction in extracellular lactate levels, which impaired neuronal energy metabolism and function [178]. Second, the cellular response of astrocytes to Tat is a metabolic shift from aerobic glycolysis to mitochondrial respiration mediated by the mitochondrial Ca2+ uniporter (MCU) regulating Ca^2+^ uptake. Targeting MCU has been found to rescue glycolysis and normalize extracellular lactate levels in astrocytes [178]. Third, astrocyte cellular stress mediated by Tat utilizes fatty acids as the energy source to support mitochondrial respiration [178]. Finally, HIV Tat impacts oxidative injury in astrocytes by reducing glutathione synthase. These changes in turn activate AMPK and increase glycolytic enzymes along with oxidative phosphorylation [179].

Neurological symptoms are more commonly observed in patients with severe acute respiratory syndrome coronavirus 2 (SARS-CoV-2; family *Coronaviridae*) infection. A number of studies have indicated that the foci of SARS-CoV-2 infection are also observed in astrocytes. The study shows that infected astrocytes alter key proteins and metabolites involved in glycolysis, gluconeogenesis, and the PPP. The decrease in lactate and pyruvate was due to the induction of oxidative metabolism in infected cells [180]. To supply the metabolic intermediates used in oxidative phosphorylation, infected astrocytes tend to alter other metabolic pathways, including glutamine metabolism. Intriguingly, among glutamine metabolic intermediates, glutamine, glutamate, and gamma-aminobutyric acid (GABA) are gradually decreased following SARS-CoV-2 infection [180, 181]. As astrocytes cooperate with neurons to maintain the glutamate-glutamine cycle in the brain, a reduction in glutamine metabolism in astrocytes may shape brain activities.

## Virus Infection Affects Neurotransmission System

Previous reports have demonstrated the involvement of viral infection in neuronal dysfunction by affecting neurotransmitter systems. Neurotransmitters are endogenous chemical messengers by which neurons communicate with each other to enable the brain to regulate a variety of functions through the process of synaptic transmission. Alterations in neurotransmitter levels have been observed in some neurotropic viral infections and are correlated with the impairment of specific brain functions. Dopamine is the main catecholamine neurotransmitter that controls voluntary movement, cognition, and endocrine regulation [182]. JEV infection significantly increases dopamine production and modulates the rate-limiting enzyme of dopamine biosynthesis, with an increase in phosphorelated tyrosine hydroxylase levels at the early stage of infection [183]. JEVs exploit dopamine-mediated neuronal communication to increase the susceptibility of dopamine D2 receptors (D2R)-expressing cells to JEV infection, which causes damage to dopaminergic neuron-rich areas such as the thalamus and midbrain, leading to neuronal loss and increasing the fatality rate of JEV-infected mice [183]. The marked decline in catecholamine levels, including norepinephrine, dopamine, 3,4-dihydroxyphenylacetic acid, homovanillic acid, and serotonin, which are unrecoverable, has been reported in the brain, and it leads to the impairment of locomotor activity in JEV-infected rats [184]. The cholinergic system plays an important role in cognitive function. The decrease in acetylcholinesterase (AChE) activity together with damage to different brain regions is associated with the transient dysfunction of learning ability in JEV-infected rats [185, 186]. The decline in muscarinic cholinergic signaling in several brain regions of JEV-infected rats includes the expression levels of cholinergic receptor muscarinic 2 and choline acetyltransferase, and the reduction in total muscarinic cholinergic binding is correlated with transient spatial learning and memory impairment [186].

The balance of glutamate, the most abundant excitatory neurotransmitter, and GABA, an inhibitory neurotransmitter in the CNS, plays a crucial role in several brain functions, including synaptic signaling, cognition, pain, and motor stimuli [187–189]. HIV infection causes the imbalance of these two neurotransmitters and is correlated with neuronal and glial dysfunction as well as cognitive impairment in the brains of HIV-seropositive patients with prolonged antiretroviral treatment [190–193].

Glutamate-mediated excitohypertoxicity is an important mechanism of neuronal injury by viral infection. Several studies have revealed the effect of HIV-1 proteins on neurotoxicity induced by glutamatergic system dysregulation [194]. The HIV-1 Tat protein induces the formation of a macromolecular complex involving N-methyl-d-aspartic acid (NMDA) receptors that promotes apoptosis in neurons and astrocytes [195–197]. Dysregulation of glutamate can contribute to HIV-associated neurocognitive disorder [198, 199]. Similar to JEV infection, the increase in glutamate-mediated excitotoxicity activity is correlated with the increase in oxidative stress observed in the brain region responsible for memory and learning impairment in JEV-infected animals [200]. The increased glutamate levels and decreased levels of its NMDA receptors are associated with the activation of TNF-α signaling and the presence of redox imbalance in neural cells, which promote neuronal death [200, 201]. These results suggest that neurotropic virus infection can alter the neurotransmission systems that lead to the dysregulation of brain function and contribute to virus-induced neuropathogenesis.

## Virus Infection Accelerates Alzheimer's Disease-Like Pathological Features

Alzheimer’s disease (AD) is the most common cause of dementia that leads to the death of elderly persons. Although age is the most important risk factor for the AD, however, several infections have been suggested to increase the risk of AD. AD is characterized by progressive impairment of synaptic function and degeneration in the brain. Two major pathological features associated with AD include amyloid and tau aggregates in the brain. Amyloid beta (Aβ) plaque accumulation is the main hallmark observed in the brains of AD patients [202]. Aβ is produced by the proteolytic process of the amyloid precursor protein (APP) that occurs at biological membrane. The cleavage of APP by α-secretase or enzymes from the disintegrin and metalloproteinase domain proteins (ADAM) family occurs within the Aβ region on APP; therefore this pathway does not produce Aβ peptide in neurons and called nonamyloidogenic pathway [203]. After α-secretase cleavage, soluble APP α (sAPPα) and α-C-terminal fragment (α-CTF, CTF-83 or C83) are generated. The α-CTF is further cleaved by γ-secretase complex releasing small fragment of extracellular p3 and the amino-terminal APP intracellular domain (AICD) which is rapidly degraded. In nonamyloidogenic pathway, the AICD is rapidly degraded [204]. On the other hand, the amyloidogenic pathway initiates with the cleavage of APP by β-secretase or β-site APP-cleaving enzyme 1 (BACE1), releasing the sAPPβ and leaving the β-C-terminal fragment (β-CTF, CTF-99 or C99) at the membrane. The processing of γ-secretase in this pathway generates two protein products including Aβ and AICD, which are different from another pathway by acting as transcriptional regulators of several target genes. Because γ-secretase can cleave many positions within CTF99, it can create different lengths of Aβ peptide, and the predominant forms are Aβ_42_ and Aβ_40_. The insoluble extracellular Aβ aggregates into plaques and can accumulate in the brain [205].

Another neuropathological feature of AD is the accumulation of neurofibrillary tangles (NFTs). NFTs are the result of hyperphosphorylation of the microtubule-stabilizing protein tau. Tau protein is highly expressed in neuronal cells and plays a role in axonal microtubule stabilization, modulation of axonal transport, and neuronal polarity. Tau phosphorylation occurs at serine and threonine sites that are regulated by balancing multiple kinases, such as glycogen synthase kinase 3 beta (GSK-3β) and cyclin-dependent kinase-5 (Cdk-5). Excessive kinase and reduced phosphatase activities cause hyperphosphorylated tau to detach and self-aggregate and cause microtubules to become destabilized [206]. Moreover, GSK-3β, a protein kinase, is not only involved in tau hyperphosphorylation but also linked to other aspects of AD pathogenesis, including Aβ production and mitochondrial dysfunction [207]. Tau deposition in the brain has been suggested to be the consequence of Aβ plaque accumulation [208]. The synergy of Aβ and NFT accumulation is distributed to various neurodegenerative mechanisms, including cholinergic degeneration, synaptic impairment, and neuroinflammation, which are partially responsible for cognitive and behavioral deficits [209].

As demonstrated in several studies, viral infections are related to the induction of pathological hallmarks of AD in association with neuronal damage, synaptic dysfunction, and impairment of cognitive functions. Most of the studies have shown the link between latent viral infections and the risk of developing AD. Latent infections from viruses such as HSV and HIV cause long-term activation of the immune system that leads to chronic neuroinflammation and neurodegeneration in the CNS [210–212]. Previous observations reveal a possible indication of HIV and HSV infections as a risk factor associated with AD by distribution of APP and tau-processing homeostasis [213, 214].

The presence of high levels of HSV and its genome have been found in brain tissues, especially in the frontal and temporal cortices and in the CSF of AD patients [211, 215–219]. HSV-1 is a neurotropic virus that lives long within the host and may reactivate after latency and penetrate the BBB into the limbic system and other brain areas that are most often affected in AD [218, 220]. HSV-1 infection causes the alteration of APP processing and has potential causality in the involvement in AD pathogenesis. HSV-1 induces multiple cleavages of APP and promotes intracellular accumulation of various neurotoxic species of Aβ [221–223]. The upregulation of BACE1 and the γ-secretase subunit (nicastrin) in the cultured neuronal and glial cells after HSV-1 infection indicates the induction of the amyloidogenic pathway by HSV-1, which leads to a dramatic increase in the intracellular levels of Aβ_40_ and Aβ_42_ [224] and in the autophagic compartments [223]. HSV-1 infection modulates the process of autophagy, as shown by the failure of the fusion of autophagosomes containing Aβ with lysosomes, indicating the impaired degradation of Aβ localized in the autophagic vesicles, which contributes to the accumulation of Aβ characteristic of AD [223]. Moreover, HSV-1 infection is associated with the inhibition of the nonamyloidogenic pathway by decreasing α-secretase activity in HSV-1-infected neuroblastoma cells [223]. Oxidative stress induced by HSV-1 potentiates the accumulation of intracellular Aβ and further inhibits its extracellular secretion, which disrupts the autophagic flux in the infected cells [225]. Furthermore, HSV-1 causes functional changes in cortical neurons that induce activity- and Ca^2+^-mediated APP phosphorylation and intracellular Aβ production [226]. Aβ protein deposits are present in the brains of mice infected with HSV-1 and show cognitive impairment in recurrent HSV-1-infected mice [224, 227]. The induction of APP amyloidogenic processing by HSV-1 also leads to the accumulation and nuclear translocation of AICD in HSV-1-infected neuronal cells [228]. This increase in AICD products in neurons has been observed to bind at the promoter and regulate the transcription of the neprilysin (*NEP*) gene, whose products are involved in the Aβ clearance process at early stages of infection. In addition, AICD regulates the expression of the *GSK3β* gene, which plays a role in tau phosphorylation during the late stage of HSV-1 infection [228]. Moreover, the increase in intracellular Aβ accumulation and GSK-3 activation induces synaptic dysfunction, as observed by the reduction in presynaptic proteins (i.e., synapsin-1 and synaptophysin) and the depressed synaptic transmission in cultured cortical neurons infected with HSV-1 [229]. HSV-1 infection affects tau processing by increasing the kinetics of tau aggregation and NFT formation. In the brains of HSV-1-infected mice, p-tau and its cleaved fragments are significantly increased in the cortex and hippocampus compared with noninfected mice [227, 230]. Infection with HSV-1 also induces an increase in tau cleavage, a marker of early neurodegeneration, by the activation of caspase-3 activity in neural cells, which indicates the involvement mechanism of HSVs in the alteration of tau processing homeostasis [227, 231]. In addition, HSV-1 reactivations cause hyperphosphorylation of tau at several sites, including serine 202, threonine 212, serine 214, serine 396, and serine 404, by the induction of GSK-3β and protein kinase A (PKA) activity [229, 231–233]. The HSV-1 protein can activate the PI3K/AKT signaling pathway to facilitate viral infection, protein synthesis, and reactivation [234, 235]. The PI3K/AKT pathways are known to regulate GSK-3 activation, which is involved in several cellular functions, including APP and tau hyperphosphorylation [236, 237]. The PI3K/AKT/GSK-3 pathways may be a crucial mechanism involved in the progressive accumulation of AD pathological hallmarks in HSV-1 infection. In the case of herpes simplex virus type 2 (HSV-2) infection, HSV-2 causes a marked accumulation of Aβ_40_ and Aβ_42_ in human SK-N-MC neuroblastoma cells [238]. HSV-2 infection decreases the levels of secreted APPα and α-CTF, which indicates the disruption of the APP nonamyloidogenic pathway [238]. Moreover, HSV-2 also induces tau phosphorylation in neuronal cells [238]. Latent infection with HSVs poses a risk of developing AD pathological features in the brain [211].

Epidemiologic studies have implicated the possible involvement of varicella zoster virus (VZV) in AD/dementia [239]. VZV (family *Orthoherpesviridae*) is an exclusively human neurotropic alphaherpesvirus that causes varicella (chicken pox) upon primary infection after which the virus establishes latency in ganglionic neurons along the entire neuraxis and the reactivation causes zoster (shingles) [240]. VZV causes gliosis and increased levels of several proinflammatory cytokines in human-induced neural stem cells (hiNSC). VZV infection of hiNSCs quiescently infected with HSV-1 leads to HSV-1 reactivation, and the increases in Aβ and p-tau accumulation were observed [239]. This suggests that VZV may reside latently in the brain and, upon reactivation, cause direct damage leading to AD through induced inflammation, which leads to neuroinflammation and reactivation of HSV-1 in the brain and consequent AD-like changes [239]. This report supports the indirect role of VZV in AD/dementia via reactivation of HSV-1 in the brain.

In HIV infection, Aβ deposition is one of the pathologic features found in the brains of HIV patients with prolonged antiretroviral treatment and aging [241, 242]. In long-term HIV infections, the alteration of the accumulation of Aβ_42_, total tau, and p-tau levels in CSF of HAND cases is similar to that of AD patients [243]. HIV viral proteins may be continually produced, increasing the risk of aged patients developing AD. HIV-1 Tat protein induces Aβ accumulation, tau phosphorylation, and subsequent neuronal death, causing slow cognitive and motor movements, seizures, and premature death [244–247]. HIV-1 Tat protein reduces clearance of Aβ_42_ from the brain to the blood and promotes nuclear entry of Aβ as well as inflammatory responses in human brain endothelial cells [248]. Furthermore, HIV-1 Tat protein enhances the cleavage of APP by β-secretase, as shown by the increased levels of β-CTF and reduced levels of α-CTF, resulting in elevated levels of Aβ_42_ in HIV-1-infected neurons [246, 249]. HIV-1 Tat protein induces impairment of endolysosome structure and function and influences the pathways of Aβ generation, degradation, phagocytosis, and transport, which contribute to HIV-1 neuropathogenesis [249, 250]. Tau processing and HIV-1 Tat protein accelerate tau phosphorylation via multiple mechanisms that lead to the formation of NFTs in HIV transgenic animals [48, 244, 250]. Tau hyperphosphorylation is found in the hippocampus of HIV-1 patients, and high levels of hyperphosphorylated tau are marked in prolonged anti-retroviral therapy-treated subjects [242]. Thus, neurotoxic viral proteins could be a risk factor for brain damage subsequent to AD and/or HIV-associated cognitive impairment.

## Viral Infection Induces Proteins to Misfold and Aggregate

The aggregation of aberrantly folded specific proteins is a pathogenic mechanism underlying several neurodegenerative diseases. The misfolded proteins are involved in neurodegenerative diseases including Aβ, p-tau, alpha-synuclein (α-syn), transactive response DNA-binding protein 43 (TDP-43), and prion protein (PrP), share common structural, biological, and biochemical characteristics, as well as similar mechanisms of aggregation and self-propagation. Virus infection induces the deposition of Aβ and p-tau that increases the risk of developing AD, which we have already discussed in the previous section.

Several neuroinvasive viruses have shown a potential relationship to develop PD. PD is a second most common neurodegenerative disorder characterized by progressive loss of dopaminergic neurons of the substantia nigra pars compacta and the presence of Lewy bodies (LBs) together with a reduction of dopamine concentration in the striatum [251]. Abnormal proteinaceous aggregates of α-syn, a major component in LBs, play a causative role in PD pathogenesis [252]. Several studies show that patients with WNV, JEV, DENV, HIV, influenza-A, hepatitis C virus (HCV), hepatitis B virus (HBV), and SARS-CoV-2 infections are at significant risk for developing Parkinson’s symptoms, including tremor, cogwheel rigidity, bradykinesia, and wide gait [253–258]. The increase in the amount of α-syn protein found in the primary neurons [259] and in the brains of patients with acute WNV encephalitis [259] and in the brains of HIV patients [260]. HIV-1 Vpr protein triggers the accumulation of α-syn in neurons [261]. Recent *in vitro* study showed that SARS-CoV-2 spike protein and nucleocapsid protein were found to accelerate the upregulation and aggregation of α-syn, which was associated with the LBs formation [262, 263].

The accumulation of intracellular ubiquitin inclusion bodies in motor neurons in the brain and spinal cord is one of the neuropathologic hallmarks of ALS. TDP-43 is a major component of inclusion bodies in pathological deposits [264, 265]. A number of reports have shown that TDP-43 plays a major role in virus entry, replication, and latency in several viruses [266]. TDP-43 proteinopathy, which is associated with neuronal dysfunction, is induced by several viral infections [266]. The TDP-43 levels were found to increase in the serum of WNV [267] and SARS-CoV-2 [268] patients in association with the high levels of inflammatory markers. Phosphorylated TDP-43 (pTDP-43) inclusion bodies have been found in the brains of SARS-CoV-2 infection [269]. TDP-43 has been shown to be potentially involved in both HIV-1 [270] as well as in HSV-2 [271] latency and cell permittivity, suggesting a possible role for TDP-43 in HIV-1 and HSV-2-associated neurodegeneration in latent infection.

Aggregation and spread of a cellular prion protein (PrP^C^) throughout the brain also play an important role in the pathogenesis of AD. PrP^C^ is converted into an aggregated neurotoxic isoform called scrapie prion protein (PrP^Sc^) that causes the neuronal death in prion diseases such as Creutzfeldt-Jakob disease in humans, which shared several neuropathological similarities links to AD [272–274]. In the hippocampus and temporal cortex of AD brains, PrP^C^ has been shown to co-localize with Aβ plaques [275, 276]. PrP^C^ functions as a receptor for Aβ_42_ oligomers and mediates the Aβ-induced synaptic dysfunction [277]. The binding of Aβ oligomers to PrP^C^ activates an Aβ-induced signaling cascade involving metabotropic glutamate receptor 5 (mGluR5), tyrosine-protein kinase Fyn, proline-rich tyrosine kinase 2 (Pyk2), and eukaryotic elongation factor 2 kinase (eEF2K) that links Aβ accumulation and tau hyperphosphorylation, resulting in the synaptic failure and neuronal death in the CNS [277–282]. PrP expression is upregulated *in vitro* by infection with a variety of viruses including HCV, HIV-1, human adenovirus type 5, Epstein–Barr virus, murine leukemia virus, and vesicular stomatitis virus [283]. Recent report shows that influenza A virus infection causes PrP^C^ to misfold into PrP^Sc^ in mouse neuroblastoma cells [284]. PrP^C^ is significantly elevated in the both neuronal cells and CSF of HIV-1-infected individuals with neurocognitive impairment and mediates neuroinflammation by inducing chemokine release by astrocytes [285]. This evidence suggests that viral infection promotes PrP misfolding and formation of infectious prions in associated with the progression of CNS neurodegeneration, particularly in the pathogenesis of AD.

Thus, viral infection is involved in several specific neurodegeneration-related protein misfolding and subsequent processes of protein aggregate propagation in the CNS leading to neuronal dysfunction, neuroinflammation, and neuronal death that contribute to neurodegenerative disease.

## Conclusion

**Fig. 3:**
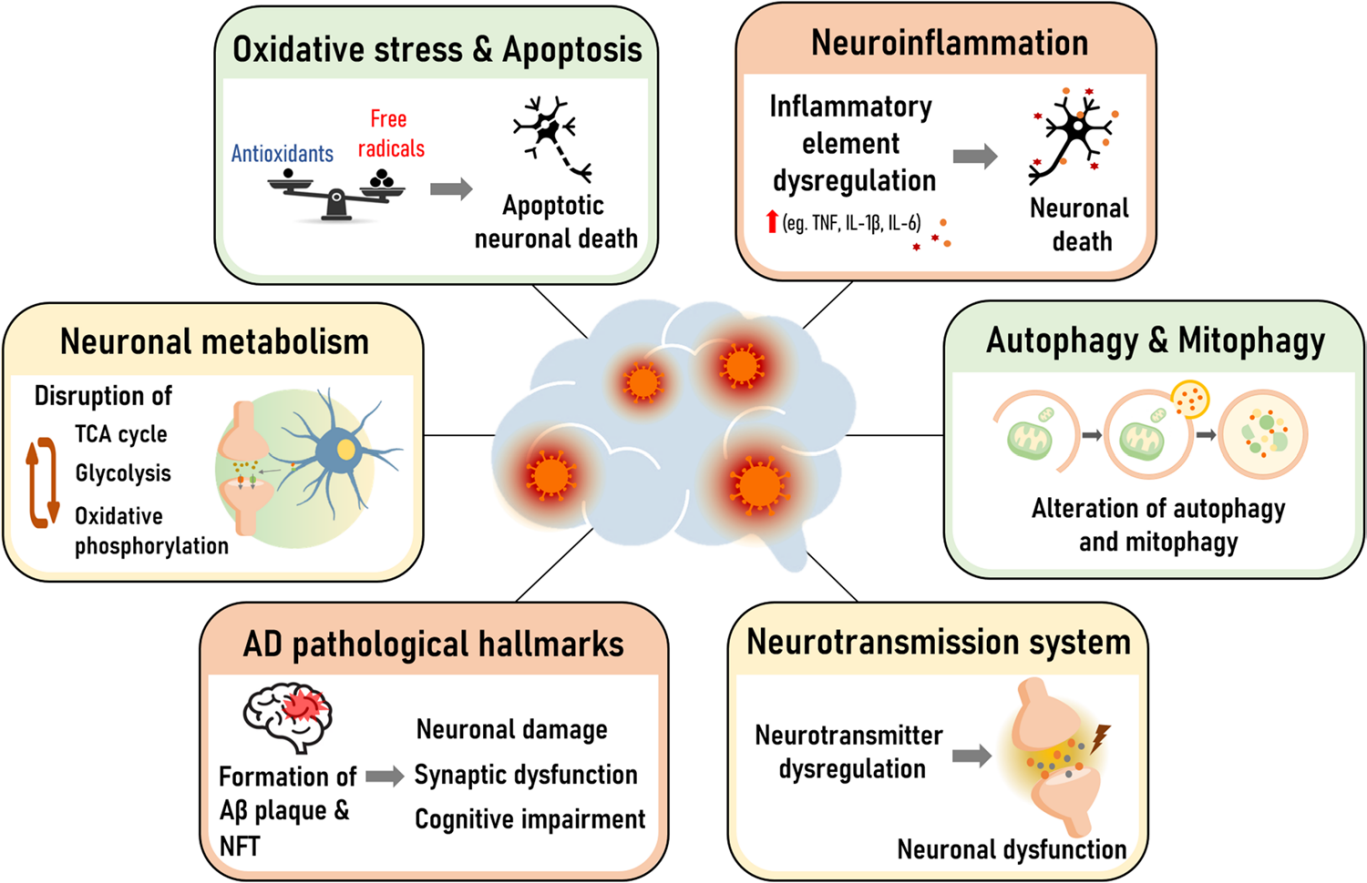
Schematic representation showing the molecular mechanisms associated with neurodegeneration of viral infection in the CNS. Abbreviation: AD, Alzheimer’s disease; Aβ, amyloid beta; NFT, neurofibrillary tangle; TCA, tricarboxylic acid

The diagnosis and treatment of CNS infection are urgent needs and challenging. Currently, there are limited or no effective antiviral drugs available for viral infection of the CNS. Neurotropic viruses can cause CNS disease through a variety of molecular mechanisms (Fig. 3), including direct or indirect immune activation, which contributes to the degeneration of neuronal cells. Viral infection promotes an imbalance between free radicals and antioxidants, which increases cellular oxidative stress and leads neuronal cells to undergo programmed cell death by apoptosis. Viruses encounter the cellular recycling process and induce impairment of mitophagy and mitochondrial dynamics in host cells. Dysregulation of mitochondrial homeostasis by viruses affects neuronal metabolism and disrupts brain function. Alterations in neurotransmitter systems and the presence of pathological hallmarks of AD can be observed in neurotropic viral infections in correlation with the specific destruction of brain functions. The fastest protection or cessation of neuronal damage by virus invasion can help to relieve symptoms, which could enhance the survivor rate and reduce severe neurological sequelae. An understanding of the neuropathogenesis of viral CNS infection could support intensive diagnosis and treatment strategies by targeting CNS molecular mechanisms and might be useful for the screening of novel antiviral agents that are essential to improving the management of these neurotropic viral infections.

## Data Availability

Not applicable

## Abbreviations

4-HNE: 4-hydroxy-2-nonenal
AchE: Acetylcholinesterase
ACOX1: Peroxisomal acyl-coenzyme A oxidase 1
AD: Alzheimer’s disease
ADAM: Disintegrin and metalloproteinase domain proteins
AICD: Amino-terminal APP intracellular domain
AIDS: Acquired immunodeficiency syndrome
ALS: Amyotrophic lateral sclerosis
ANLS: Astrocyte-to-neuron lactate shuttle
AP-1: Activator protein 1
APP: Amyloid precursor protein
ARE: Antioxidant response element
ATF4: Activating transcription factor 4
Atg: Autophagy-related gene
Aβ: Amyloid beta
BACE1: β-site APP-cleaving enzyme 1
BAX: BCL-2 associated X
BBB: Blood–brain barrier
BCL-2: B-cell lymphoma 2
Ca^2+^: Calcium ion
CAT: Catalase
CCL-2: Chemokine (C-C motif) ligand 2
CCL-5: Chemokine (C-C motif) ligand 5
CCR5: C-C motif chemokine receptor 5
Cdk-5: Cyclin-dependent kinase-5
CDS: Cytosolic DNA sensors
CHOP: C/EBP homologous protein
CHPV: Chandipura virus
CLRs: C-type lectin receptors
CNS: Central nervous system
CSF: Cerebrospinal fluid
CXCL10: C-X-C motif chemokine 10
CXCR4: C-X-C motif chemokine receptor 4
CYP2E1: Cytochrome P450-2E1
Cyt c: Cytochrome c
D2R: Dopamine D2 receptors
DENV: Dengue virus
DENV-2: Dengue virus type 2
DENV-3: Dengue virus type 3
DRP1: Dynamin-related protein 1
eEF2K: Eukaryotic elongation factor 2 kinase
EGR1: Early growth response 1
eIF2α: Eukaryotic translation initiation factor 2α kinase
ER: endoplasmic reticulum
ERK: Extracellular-signal-regulated kinase
ETC: Electron transport chain
EV71: Enterovirus 71
GABA: Gamma-aminobutyric acid
GADD153: Growth arrest and DNA damage-inducible protein
GMP: Cyclic guanidine monophosphate
GPx: Glutathione peroxidase
GR: Glutathione reductase
GRP78: Glucose-regulated protein 78 kDa
GSH: Glutathione
GSK-3β: Glycogen synthase kinase 3 beta
HAEs: Hydroxyalkenals
HANDs: HIV-associated neurocognitive disorders
HBV: Hepatitis B virus
HCV: Hepatitis C virus
HD: Huntington’s disease
HFMD: Hand, foot, and mouth disease
HIV: Human immunodeficiency virus
HSP: Heat shock protein
HSV: Herpes simplex virus
HSV-1: Herpes simplex virus type 1
HSV-2: Herpes simplex virus type 2
IFNγ: Interferon gamma
IL: Interleukin
iNOS: Inducible nitric oxide synthase
JEV: Japanese encephalitis virus
LBs: Lewy bodies
LC3B: Microtubule-associated protein 1 light chain 3 beta
MAPK: Mitogen-activated protein kinase
MCP-1: Monocyte chemoattractant protein-1
MCU: Mitochondrial Ca^2+^ uniporter
MDA: Malondialdehyde
mGluR5: Metabotropic glutamate receptor 5
MIP-1α: Macrophage inflammatory protein 1 alpha
MOI: Multiplicity of infection
MPR: Mannose-6-phosphate receptor
mTOR: Mammalian target of rapamycin
NAD+: Nicotinamide adenine dinucleotide
NADPH: Nicotinamide adenine dinucleotide phosphate
NFTs: Neurofibrillary tangles
NF-κB: Nuclear factor kappa B
NLRP3: NLR family pyrin domain containing 3
NLRs: NOD-like receptors
NMDA: N-methyl-d-aspartic acid
NMNAT2: MAPK-mediated nicotinamide mononucleotide adenylyltransferase 2
NO: Nitric oxide
NPCs: Neural progenitor cells
NRF2: Nuclear factor erythroid 2-related factor 2
NS4B: Nonstructural protein 4B
O2.-: Superoxide anions
OONO-: Peroxynitrite
p70S6K: Phosphorylated 70-kDa S6 kinase
PARP: Poly ADP-ribose polymerase
PD: Parkinson’s disease
PERK: Protein kinase RNA-like ER kinase
PET: Positron emission tomography
PINK1: PTEN-induced kinase 1
PKA: Protein kinase A
PMP22: Peripheral myelin protein 22
PPP: Pentose phosphate pathway
PrP: Prion protein
PrP^C^: Cellular prion protein
PrP^Sc^: Scrapie prion protein
PRRs: Pattern recognition receptors
pTDP-43: Phosphorylated TDP-43
Pyk2: Proline-rich tyrosine kinase 2
RABV: Rabies virus
RANTES: Regulated on activation, normal T-cell expressed, and secreted
RIG-I: Retinoic acid-inducible gene I
RLRs: Retinoic acid-inducible gene-I-like receptors
ROS: Reactive oxygen species
sAPPα: Soluble APP alpha
SARS-CoV-2: Severe acute respiratory syndrome coronavirus 2
Ser51: Serine51
SOD-1: Superoxide dismutase 1
SQSTM1: Sequestosome 1
TBARS: Thiobarbituric acid reactive substances
TBEV: Tick-borne encephalitis virus
TCA: Tricarboxylic acid cycle
TDP-43: Transactive response DNA-binding protein 43
TGF-β: Transforming growth factor beta
TLR: Toll-like receptor
TNF-α: Tumor necrosis factor alpha
TNF-β: Tumor necrosis factor beta
TRADD: TNF receptor-associated death domain
TRX-1: Thioredoxin 1
TSPO: Translocator protein
UPR: Unfolded protein response
VEEV: Venezuelan equine encephalitis virus
VP1: Viral envelope protein 1
VZV: Varicella zoster virus
WNV: West Nile virus
ZIKV: Zika virus
α-CTF: α-C-terminal fragment
α-syn: Alpha-synuclein
β-CTF: β-C-terminal fragment
